# Mental health problems among adolescents during the COVID-19 pandemic: a repeated cross-sectional study from Sweden

**DOI:** 10.1177/14034948231219832

**Published:** 2024-01-12

**Authors:** Håkan Källmen, Mats Hallgren

**Affiliations:** 1 Department of Psychology, Uppsala University, Sweden; 2 Epidemiology of Psychiatric Conditions, Substance Use and Social Environment (EPiCSS), Karolinska Institutet, Sweden

**Keywords:** Youth mental health, COVID-19, pandemic, adolescent, social support

## Abstract

**Objective:**

Most international studies have concluded that exposure to the COVID-19 pandemic was associated with worse mental health. Sweden implemented lighter restrictions than many other countries. We evaluated the association between changes in exposure of COVID-19 restrictions and changes in mental health problems among Swedish adolescents.

**Method:**

Repeated cross-sectional data were derived from the Stockholm school survey, mandatory for all students in municipal schools and voluntary for students in private schools. Unexposed students were assessed in the year 2020 and exposed were assessed in 2022. Mental health was assessed using items similar to the psychosomatic problem scale. All variables were dichotomised, and a non-parametric logistic regression was used to evaluate associations.

**Results:**

A significant positive association was found between exposure of COVID-19 restrictions and self-reports of five to seven mental health problems a week for girls (odds ratio (OR) 1.29, 95% confidence interval (CI) 1.18–1.41), but a non-significant association was found for boys (also after controlling for relevant covariates). Among boys and girls (shown), changes in mental health during the pandemic were moderated by social support from parents (OR 2.23, 95% CI 1.98–2.51), bullying victimisation (OR 2.24, 95% CI 2.06–2.66), a sensation-seeking temperament (OR 1.40, 95% CI 1.24–1.58) and school achievement (OR 1.34, 95% CI 1.18–1.51).

**Conclusions:**

**Compared with boys, adolescent girls self-reported worse mental health following COVID-19 exposure. Girls may have been more affected by social distancing regulations in Sweden during the pandemic than boys and may require additional psychosocial support post-pandemic. Social support from parents may play an important role.**

## Introduction

The COVID-19 pandemic (the pandemic) spread rapidly during the beginning of 2020. The Swedish Public Health Agency recommended restrictions on people's behaviour, primarily physical distancing, starting in mid-March 2020, which ended in March 2022 [1]. During this time, people were encouraged to use municipal transportation only for essential travel, and to avoid crowding in public places. Social distancing was encouraged. High schools and universities were closed and most teaching was done online. Although lighter restrictions were implemented in Sweden compared with some other countries, it is possible that the restrictions have detrimentally affected mental health [2].

Studies examining the association between COVID-19 restrictions and mental health in Sweden are inconclusive. Chen et al. examined the effect of the pandemic on the mental health of Swedish adolescents [3]. Longitudinal data were collected from 1900 adolescents between September 2017 and November 2020. Adolescents reported higher levels of stress and psychosomatic symptoms and lower levels of happiness at follow-up compared with baseline [3]. Another Swedish study investigated changes in youth psychosocial functioning during ‘times of crisis’ (*n*=1767 adolescents aged 15–19 years) [4]. Findings indicated that most youth self-reported negative mental health effects after 3 to 4 months of exposure to the pandemic [4]. Nyberg et al. investigated changes in mental health among 585 boys and girls aged 13–14 years during 2019 and 2021 [5]. Self-reported mental health had deteriorated during the pandemic (compared with before). A study investigating compliance with recommended COVID-19 restrictions among 4495 university students in Sweden showed that compliance rates were above 70% [6]. Compared with those who were compliant with restrictions, non-compliance was associated with more COVID-19 symptoms [6]. A cross-sectional study comparing the effects of the pandemic on adolescent psychological distress in five countries (Morocco, Serbia, Sweden, Vietnam and the USA) [7] showed that psychological distress was positively associated with perception of COVID-19 impact in four countries; however, this association was not observed in Sweden [7]. This suggests that, among psychologically distressed adolescents, the pandemic had a lesser impact in Sweden compared with other countries. Another study used the same data but focused on gender differences, and found that girls retrospectively perceived a stronger impact of the pandemic on their lives compared with boys [8].

Other factors may also impact youth mental health regardless of the pandemic, and need to be accounted for when examining these relationships. Differences in mental health problems may partly be attributable to parents’ socioeconomic status [9, 10]. A cross-sectional study of more than 33,000 Swedish students aged 15 and 18 years showed that bullying at school was associated with poor mental health [11]. Parental divorce and support are also shown to impact youth mental health [12, 13]. At the individual level, personality has been linked to mental health. One study showed that sensation-seeking and boredom were detrimentally associated with mental health among youth [14]. A negative association was shown between poor school performance and the use of psychotropic medications among Swedish youth [15]. These findings are consistent with a study by von Simson et al. (*n*=242,542 Norwegian adolescents) [16]. Alcohol and drug use has also been associated with worse mental health in previous studies [17].

The aim of this study was to evaluate the association between changes in exposure to the COVID-19 restrictions and changes in mental health problems among Swedish adolescents. We used a 2-year follow-up period, from the implementation to the end of the nationally recommended restrictions. Together with a large number of respondents this study differs from earlier research. A secondary aim was to examine gender differences [8]. Due to the lighter pandemic restrictions in Sweden compared with other countries, we hypothesise that, after controlling for relevant factors, there would be no significant association between COVID-19 exposure and mental health problems among adolescents.

## Methods

### Design

The study had a repeated cross-sectional design with two waves: 2020 and 2022. There were different participants in both waves. In each wave assessments were made on students in lower secondary school, year 9, and in upper secondary school, year 11.

### Participants

The Stockholm school survey is completed in March every other year by students in lower secondary school (year 9 – compulsory) and upper secondary school (year 11) [18]. About 91% of participants attended schools in Stockholm municipality, but about 27% were residents in other municipalities in the Stockholm metropolitan area. The survey is mandatory for municipal schools, but voluntary for private schools. The purpose of the survey is to help inform decision making by local authorities that will ultimately improve the student wellbeing [18]. The questions relate to life circumstances, schoolwork, bullying, drug use, health and crime. Non-completers are those who for some reason were absent from school when the survey was completed. Available cross-sectional data consist of responses to the survey in two waves. The unexposed students, years 9 and 11, were assessed in March 2020 at the beginning of the implementation of the restrictions, and the exposed students, years 9 and 11, were assessed in March 2022 at the end of restrictions. The majority of the data has been collected during the month of March 2020, but some schools have sent in data during April 2020. The data have thus been collected at the same time as the COVID-19 restrictions were introduced. The spread of infection during the month of March may have influenced the responses, probably in the direction of more participants reporting mental health problems at the 2020 assessment. Response rates at school level were 72% in 2020 and 77% in 2022. In 2020 there were 5633 boys and 5865 girls, and in 2022 there were 6926 boys and 7195 girls who responded to the survey.

### Procedure

In 2020 students completed the questionnaire during a school lesson and then placed it in a sealed envelope before handing it to their teacher. Students had the entire lesson (about 40 minutes) to complete the questionnaire. In 2022 half of the participants responded on a paper version and half responded to an electronic version of the survey. Students were informed that it is voluntary to participate and that they are free to withdraw their participation at any time without consequences. Students also receive information that the Origo Group, a private evaluation company, is responsible for collection of the data on behalf of the City of Stockholm [18].

### Measures

Mental health problems (primary outcome) were assessed by using a scale similar to the psychosomatic problem scale, PSP scale [19]. In the Stockholm school survey, there are 10 questions about how often students experience various psychosomatic problems. Six of these are identical to questions in the psychosomatic problems scale. Together with a question about how often the respondent feels that it is great to be alive, an index was created. This index is identical to that used in a previously published article [11]. In this study we used seven different symptoms or problems, including: headaches, depression, feeling fear, stomach problems, difficulty sleeping and poor appetite, which once or several times a week were coded 1 and never or at most once a month were coded 0. The item thinking it is great to be alive was coded negatively as seldom or any single time as 1. A principal axis factor analysis (current data) showed that a single factor with the Kaiser criterion eigenvalue above 1 could be extracted, eigenvalue 2.54. Six items loaded between 0.48 and 0.65 on this factor. Depression loaded highest and feeling fear loaded below 0.40. On some items the response scale was from never to very often, but on others it was from never to several times a week. If students responded (on a five-point scale) that any of above problems occurred rather or very often or at least once a week during the previous year it was seen as an indicator of a possible mental health symptom and coded 1. Internal consistency was fair, Cronbach alpha 0.70. By adding these problem areas, a total index is created from zero to seven mental health symptoms. Between zero and four symptoms per week are considered as a low indication of mental health problems and coded as 0, while between five and seven symptoms are considered as an indication of a mental health problems and coded 1.

COVID-19 exposure: To estimate the influence of COVID-19 restrictions a dichotomised variable was created. The 2020 assessment was conducted before the implementation of restrictions and was coded 0, the 2022 assessment was conducted at the end of the restriction period and was coded 1.

### Covariates shown to be associated with mental health

#### Socioeconomic covariates

Housing segregation: It is generally known that the physical environment of the inner-city housing is (on average) better, incomes are higher and the ethnic segregation lower compared with the suburbs. Those factors are all risks for inequal spread of COVID-19 [2]. An item asking about which part of Stockholm respondents lived in assessed segregation. Stockholm metropolitan area was divided into 16 different parts and suburbs were coded 1 and inner city was coded 0. Socioeconomic status is usually assessed as respondent’s income and education. Due to low variation in education among students we used parents’ education as an approximation and income was operationalised as the amount of pocket money per month that the student receives for pleasure. For the variable mother’s education, if the mother did not have a university education, 1 was coded or else 0 (also for fathers). For the variable pocket money, if the student receives less than SEK 1000, approximately $120, 1 was coded or else 0. Living with both parents was assessed by an item asking about which persons the respondent lived together with. There were 10 different response alternatives and if the respondent ticked both mother and father 0 was coded or else 1. Social support from parents: Eight statements described the adolescent’s relationship with their parents. A principal axis factor analysis on the current data showed that the eight statements constituted two factors with eigenvalues above 1.0 explaining 56.4% of the covariance. See supplemental material for a description.

#### Personality-related covariates

Sensation-seeking: The Stockholm school survey contains 21 statements about attitudes and behaviours in different situations. In order to divide the statements into categories, an explorative principal axis factor analysis resulted in five factors, which together explained 53.4% of the covariance. See supplemental material for a description.

#### School-related covariates

Bullying at school was assessed by one item asking if the student felt bullied or harassed at school during the previous year. Response alternatives were ‘No’ or a list of seven possible ways to be bullied. These seven are: (a) I have been mocked, ridiculed, teased or called nicknames in an unpleasant and hurtful way; (b) I have been ostracised by other students; (c) I have been hit, kicked, pushed or locked inside; (d) Some student has spread lies or false rumours about me and tried to get others to dislike me; (e) I have been deprived of money or things or had things destroyed; (f) I have been threatened or forced to do things I did not want to do; (g) Teachers have psyched me out or otherwise been mean to me. If at least one of these ways was ticked the respondent was considered as have felt bullied and 1 was coded, if the respondent ticked ‘No’, 0 was coded. Grades in school in the participant’s Swedish, English, and mathematics class were self-reported and measured on a seven-point scale on which 1 was coded if the teacher had not been able to assign a grade for the student, F was coded as 2, E was coded as 3, D was coded as 4, C was coded as 5, B was coded as 6 and A was coded as 7. Grades A, B and C comprised more than half of the students in each subject and were considered good grades and coded 0 or else 1.

#### Substance-related variables

Nicotine use: Two questions addressed nicotine use: ‘Do you smoke?’ and ‘Do you use snus?’. For both questions, five possible answers were provided: ‘No, never’, ‘No, just tasted’, ‘No, I have previously, but stopped’, ‘Yes, occasionally but not daily’ and ‘Yes, daily’. ‘Yes, occasionally but not daily’ and ‘Yes, daily’ were coded as 1, and the remaining options were coded as 0. Alcohol use was measured by asking how often the student, on a single occasion, consumed an amount of alcohol equivalent to or more than: 18 cl of liquor, a whole bottle of wine, four large bottles of cider greater than 3.5%, four cans of greater than 3.5% beer, or six cans of 2.25–3.5% beer. Seven possible answers were provided, ranging from ‘I don’t drink alcohol’ to ‘Once a week’. ‘Once a year’ and more seldom was coded as 0, and ‘Once a month’ or more often was coded as 1. Cannabis use was measured using the following question: ‘How many times have you used hash/marijuana?’. Seven possible answers were provided, ranging from: ‘Never’ to ‘More than 50 times’. By using median-split less than five times was coded as 0, and five times or more was coded as 1.

### Statistical analyses

Descriptive statistics for estimating the proportion of girls and boys who reported mental health problems are presented and 95% confidence intervals (CIs) were calculated using the formula ± square root (*P*(1-p)/n) * 1.96. As few of the variables used were normally distributed, we dichotomised all variables and used a non-parametric logistic regression analysis to evaluate associations. Both crude and adjusted models were calculated. We also stratified all data and evaluated the gender-specific associations between mental health problems and nearly 2 years’ exposure of COVID-19 restrictions when adjusting for relevant covariates in a multivariable logistic regression analysis. The significance level was set to 0.05. To evaluate the interaction between exposure and other covariates, interaction terms were created by multiplying covariates with exposure. By using data for boys and girls combined a test of interaction between covariates and exposure was evaluated. The associations are presented as effect sizes, odds ratios (ORs) and 95% confidence intervals (CIs). Non-responses on items included in index were coded as 0. Non-responses on single items resulted in deletion of the case.

## Results

Between spring 2020 and spring 2022 the prevalence of students reporting five to seven mental health problems in the population of adolescent girls from schools in Stockholm showed a non-significant increase from 15.8 to 18.8 percentage points, and among boys there was a non-significant reduction in prevalence from 4.4 to 4.0 percentage points. To evaluate the association with COVID-19, a bivariable regression analysis was calculated on data from 2020 and 2022 combined. Table I shows frequencies and percentages for included variables stratified by gender and year. In total, 12,911 boys and 13,680 girls responded to the items. The crude model showed a weak but significant association between COVID-19 exposure and reports of five to seven mental health problems a week for girls (OR 1.29, 95% CI 1.18–1.41). The corresponding association for boys was not significant (OR 0.94, 95% CI 0.80–1.12). Table II shows the results after controlling for relevant covariates. After removing missing covariate data, there were 8017 boys and 9418 girls. Among boys, the OR for self-reported mental health problems after COVID-19 exposure increased from 0.94 to 1.04 but neither association was statistically significant. The corresponding OR for girls was higher in both survey years and remained significant; however, the change was negligible (1.29 to 1.30). After control for relevant covariates low social support from parents, sensation-seeking, bullying victimisation at school and poor grades in mathematics were significantly associated with reports of mental health problems for both boys and girls. However, a test of interaction showed that only social support and bullying moderated the association between COVID-19 exposure and mental health problems for boys and all four covariates moderated the association for girls. In addition, father’s education, living together with only one parent, binge drinking of alcohol at least once a month, having used cannabis (frequency is specified above), and grades in both Swedish and English were significant moderators for girls only. For boys only, cigarette smoking daily or almost daily moderated the association.

**Table I.** Frequencies and percentages for variables included (*N*, %).

Variable	Boys 2020	Boys 2022	Girls 2020	Girls 2022
Mental health problems	275 (4.4)	299 (4.0)	1011 (15.8)	1460 (18.8)
Living in suburbs	3560 (57.0)	3701 (48.9)	3760 (58.8)	4107 (52.9)
Mother has no university education	1161 (18.6)	1213 (16.0)	1327 (20.7)	1444 (18.6)
Father has no university education	1374 (22.0)	1403 (18.5)	1482 (23.2)	1681 (21.8)
More than 1000SEK/month for own use	2953 (47.2)	3685 (48.7)	2839 (44.4)	3566 (45.9)
Not living with both parents	1580 (25.3)	1641 (21.7)	1643 (25.7)	2032 (26.2)
Poor support from parents	3334 (53.3)	3947 (52.2)	3093 (48.4)	4052 (52.2)
High sensation-seeking	3370 (53.9)	3727 (49.2)	2927 (45.8)	3460 (44.6)
Bullied at school	790 (12.6)	1122 (14.8)	1204 (18.8)	1569 (20.2)
Poor grades in Swedish	2375 (38.0)	2504 (33.1)	1306 (20.4)	1544 (19.9)
Poor grades in English	1377 (22.0)	1315 (17.4)	1139 (17.8)	1309 (16.9)
Poor grades in mathematics	2739 (43.8)	2867 (37.9)	2921 (45.7)	3348 (43.1)
Smokes cigarettes	775 (12.4)	624 (8.2)	1191 (18.9)	1153 (14.2)
Uses snus (smokeless tobacco)	1178 (18.8)	779 (10.3)	469 (7.3)	110 (1.4)
Cannabis use	1151 (18.4)	1020 (13.5)	860 (13.4)	878 (11.3)
Binge drinking more than once a month	1132 (18.1)	1325 (17.5)	1462 (22.9)	1872 (24.1)

**Table II.** Logistic regression analysis of the association between COVID-19 exposure and mental health problems adjusted for relevant co-variates.

Variable name	Boys		Girls	
	OR	95% CI	OR	95% CI
Crude model				
Exposure for COVID-19	0.94	0.80–1.12	1.29	1.18–1.41
Adjusted model				
Exposure for COVID-19 restrictions	1.04	0.82–1.32	1.30	1.16–1.46
Socioeconomic covariates				
Living in suburbs	1.16	0.92–1.48	0.99	0.89–1.11
Mother has no university education	1.17	0.86–1.61	0.95	0.82–1.10
Father has no university education	0.83	0.61–1.12	1.16	1.02–1.34
Less than SEK1000/month for own use	1.05	0.82–1.34	1.06	0.95–1.20
Living together with only one parent	1.23	0.94–1.61	1.18	1.04–1.34
Low social support from parents	2.18	1.67–2.65	2.23	1.98–2.51
Substance-related covariates				
Smoke tobacco every or almost every day	1.67	1.16–2.40	1.08	0.92–1.28
Use tobacco-based snus every or almost every day	1.10	0.78–1.54	1.28	0.98–1.67
Binge drink alcohol at least once a month	0.91	0.64–1.28	0.83	0.71–0.97
Have used cannabis more than four times in life	1.23	0.88–1.71	1.50	1.25–1.80
Personality-related covariate				
Sensation-seeking attitude	1.44	1.10–1.89	1.40	1.24–1.58
School-related covariates				
Bullying victimisation	3.52	2.72–4.57	2.34	2.06–2.66
Low grades in Swedish	1.24	0.94–1.65	1.24	1.05–1.46
Low grades in English	0.86	0.60–1.23	0.79	0.66–0.95
Low grades in mathematics	1.59	1.22–2.06	1.34	1.18–1.51

OR: odds ratio; CI: confidence interval.

## Discussion

We examined the association between COVID-19 ex-posure and self-reports of five to seven symptoms of mental health problems weekly among adolescent girls and boys in Sweden. The crude model showed a significant bivariable association among girls but not among boys. However, during the 2-year pandemic, other factors occurring simultaneously could have moderated the association with reported mental health problems. After controlling for relevant covariates, the low but significant detrimental association between COVID-19 and reports of mental health problems remained for girls but was eliminated (non-significant) among boys. Non-responses on the covariates and a lower number included in the analysis in the adjusted model did not attenuate the main results. Our findings are comparable to those reported earlier showing that girls reported themselves to be more anxious, depressed and have more problems with sleep during the pandemic than boys [8]. This is possibly due to the physical distancing. Consistent with earlier research, social support from parents and bullying victimisation at school moderated the association [11]. This may be due to parental support becoming more important when socialising with friends is restricted. Sensation-seeking and grades in mathematics were shown to moderate the association for girls only. This may be due to the fact that girls are generally less sensation-seeking compared with boys. These findings suggest that both external factors (social support and bullying victimisation) and personality factors (sensation-seeking) need to be targeted in the treatment of mental health problems.

Among girls, poor school achievement in Swedish, English and mathematics was associated with men-tal health problems, but among boys, only grades in mathematics moderated the COVID-19 association with mental health problems. This finding is consistent with a review concluding that school achievement is affected by the socialisation process [20]. For girls, five other factors also moderated the association, namely fathers’ education, living together with only one parent, binge drinking of alcohol at least once a month and the use of cannabis (more than four times ever). These results confirm earlier research showi-ng that internalising problems, such as anxiety and depression, were more prevalent among girls than boys who lived together with only one of their parents and may be due to less support [12]. Consistent with earlier studies, social support from parents moderated the association between COVID-19 exposure and mental health, which means that a good and trustful relationship with parents or other caregivers is important for mental health; potentially more so during the pandemic [12, 13]. Bullying victimisation at school was shown also to moderate the association confirming an earlier study of bullying and adolescent mental health [11]. The association between girls’ mental health problems and cannabis use is consistent with a study showing worse mental health among adolescents using drugs [17]. Regarding the negative association between binge drinking and mental health problems among girls, this may be explained by the fact that girls with poor mental health are binge drinking alcohol less often compared with girls that do not have mental health problems [21]. As Sweden had no lockdown and a less stringent monitoring of the population’s compliance with the recommendations, the weak but significant association between exposure to COVID-19 and mental illness is consistent with previous research [22, 23].

Our study contributes new knowledge with its repeated cross-sectional design, and by controlling for several factors that may have affected participants’ mental health during the pandemic. Data were derived from a large cohort of students in Stockholm with a similar age and gender distribution. At the school level, response rates were high but somewhat lower in 2020 (due to the pandemic). The survey provides detailed information on possible confounders, and the respondents were anonymous which may reduce response bias. Some potential weaknesses are acknowledged: the cross-sectional design prohibits directionality from being established, and the cohorts assessed in each year comprised different student populations. This made it possible to evaluate changes on a group level only. As the assessments were made 2 years apart, it is possible that external factors that were not assessed, such as war in the Ukraine and the increasing climate anxiety, may have influenced the responses.

## Conclusions

Exposure to the pandemic was associated with detrimental changes in mental health among adolescent girls in Sweden. Boys appear to have been less affected by the social distancing and other measures imposed during the pandemic. These outcomes could reflect girls’ stronger reliance on social support as a coping mechanism during stressful life events (i.e. the pandemic) compared with boys. Our findings suggest that girls may require additional psychosocial support in the months and years following the pandemic. Social support from parents and bullying victimisation are factors that moderate these relationships and have strong associations with youth mental health. Future studies should examine the long-term effects of the pandemic on adolescent mental health.

## Supplemental Material

sj-docx-1-sjp-10.1177_14034948231219832 – Supplemental material for Mental health problems among adolescents during the COVID-19 pandemic: a repeated cross-sectional study from Sweden Supplemental material, sj-docx-1-sjp-10.1177_14034948231219832 for Mental health problems among adolescents during the COVID-19 pandemic: a repeated cross-sectional study from Sweden by HÅKAN KÄLLMEN and MATS HALLGREN in Scandinavian Journal of Public Health
