# Supplementary material for: Mental health problems among adolescents during the COVID-19 pandemic: a repeated cross-sectional study from Sweden
Source: Scand J Public Health. 2024 Jan 12;52(3):329–35. doi: 10.1177/14034948231219832 (PMC11067385; doi:10.1177/14034948231219832)
Supplement: sj-docx-1-sjp-10.1177_14034948231219832 – Supplemental material for Mental health problems among adolescents during the COVID-19 pandemic: a repeated cross-sectional study from Sweden [file sj-docx-1-sjp-10.1177_14034948231219832.docx]

**Supplementary material**

**Description of the factor analysis used in this study:**

A principal axis factor analysis of eight statements about respondent´s relationship to their parents was conducted. Factor 1, called parental support, explained 40.2 percent consisted of five statements loading above 0.5 on parental support. Those statements were 1. They give me praise when I do something well. 2. They usually encourage and support me. 3. They notice when I do something good. 4. I care what my parents say. 5. My parents are role models for me. The internal reliability was for this scale high, Cronbach’s alpha 0.84. The responses to each statement should be made on a four-point Likert scale scored from 1 to 4 and an expected total score from 5 to 20. Due to non-responses on certain statements the observed total score on the 5 statements ranged from 1 to 20 and was dichotomized using median split into low support, 1-16, coded 1 and high support 17-20, coded 0.

A principal axis factor analysis of 21 statements describing respondents’ attitudes and behaviours was conducted. The main factor explaining 27.2 percent of the co-variance consisted of six items with sensation-seeking content. The respondent was asked to estimate on a four-point scale to what extent the statement was true for them. The responses were 1. fits very poorly 2. Fits rather poorly 3. Fits rather well 4. Fits very well. The six statements were 1. I like to do exciting and dangerous things even if it is forbidden 2. I am often out-doors together with friends on nights 3. I ignore rules that prevent me from doing what I want to do 4. I look at myself as a rather impulsive person 5. I want to be there where exiting things happens 6. I do stupid things even if they are little dangerous. These six items constitute an index with high internal consistency, with Cronbach’s alpha of 0.82. The total score on the index was expected to range between 6 and 24, but due to non-responses on certain items the observed total score ranged between 1 and 24 and dichotomized using a median split.
